# Optimization and Development of Ready to Eat Chocolate Coated Roasted Flaked Rice as Instant Breakfast Food

**DOI:** 10.3390/foods10071658

**Published:** 2021-07-18

**Authors:** Shiv Kumar, Poonam Baniwal, Gulzar Ahmad Nayik, Kamlesh Prasad, Khalid Ali Khan, Hamed A. Ghramh, Harish Kumar, Ioannis Konstantinos Karabagias

**Affiliations:** 1 Department of Food Engineering and Technology, Sant Longowal Institute of Engineering & Technology, Longowal 148106, India; shivk1999@gmail.com (S.K.); pbaniwal@gmail.com (P.B.); 2 Department of Nutrition & Dietetics, Chandigarh University, Mohali 140413, India; 3 Department of Food Science & Technology, Government Degree College, Shopian 192303, India; gulzarnaik@gmail.com; 4 Research Center for Advanced Materials Science (RCAMS), King Khalid University, P.O. Box 9004, Abha 61413, Saudi Arabia; khalidtalpur@hotmail.com (K.A.K.); hamedsa@hotmail.com (H.A.G.); 5 Unit of Bee Research and Honey Production, Faculty of Science, King Khalid University, P.O. Box 9004, Abha 61413, Saudi Arabia; 6 Biology Department, Faculty of Science, King Khalid University, P.O. Box 9004, Abha 61413, Saudi Arabia; 7 Amity Institute of Biotechnology, Amity University Rajasthan, Jaipur 303002, India; harishkanwar3@gmail.com; 8 Department of Chemistry, Laboratory of Food Chemistry, University of Ioannina, 45110 Ioannina, Greece

**Keywords:** coating, flaked rice, morphology, sensory, response surface methodology (RSM)

## Abstract

The present study aimed to optimize and develop ready-to-eat rice-based functional breakfast food using response surface methodology. The levels of ingredients viz. skim milk powder, guar gum, and ferrous sulfate were pre-optimized and remained constant, whereas jaggery and dark chocolate were taken as independent variables. The optimum levels of jaggery and dark chocolate for chocolate-coated roasted flaked rice (CCRFR) were 8.49 g and 25.43 g, respectively. The physical, pasting, textural, functional, morphological, optical, and sensory characteristics of CCRFR and uncoated roasted flaked rice (RFR) were also studied. CCRFR had significantly higher mineral (iron and calcium) and total polyphenolic contents. Furthermore, the dimensional, sensory, and functional properties were also improved. The changes in morphological structure were also observed between the CCRFR and uncoated product using scanning electron microscopy. The coating adds nutritional value to the roasted rice and renders it an essential functional RTE convenience gluten-free cereal breakfast item.

## 1. Introduction

Paddy (*Oryza sativa* L.) is the principal cultivated crop in Asia. Being the second-largest paddy-producing country, India contributes one-fifth of the world paddy production, which amounts to 172.58 MMT in 2017–2018 out of 44.50 million hectares of cropped area [1]. Availability of rice made it a regular staple foodstuff item of the county and different ready-to-eat (RTE) rice-based food savors are, thus, also found readily available locally. Rice as an invaluable carbohydrate alternative is readily digestible and rarely induces allergic reactions [2]. Moreover, rice is considered an exceptional medium for the preparation of fortified products and nutraceuticals because of its inert taste. Furthermore, it acts as a good conveyor for the development of aromas, colors, and flavors [3].

Flaked (*Poha*) or flattened rice is traditional and popular food/snack in Asian countries [4]. Flaked rice is a good source of minerals, carbohydrates, vitamins, and antioxidants [5]. It has more rice bran oil as evaluated in white rice, which is a good source of natural antioxidants, i.e., γ-oryzanol. The function of γ-oryzanol in lowering blood cholesterol in hyper-cholesterolemic persons is well recognized [6]. The flaked rice products developed from whole rice have anti-cancerous properties in colon cells [7]. Flaked rice is already consumed as a breakfast food or snack item after soaking, roasting, frying, or garnishing with wet and dry spices [8].

Roasting flaked rice is the conventional cooking routine performed for a short time at high temperature with heat transfer through either common salt or fine sand. When roasting at an optimum combination of time and temperature, the results are the formation of better products with crispiness and flavor. Starch molecules are gelatinized during the roasting process, some part of the crystalline structure of amylopectin is transformed into an amorphous structure, and all this results in the development of resistant starch. Resistant starch molecules act as dietary fiber and decrease the glycemic index in blood, which may help to control diabetes and LDL levels [9]. Apart from helping in bowel movement and reducing constipation problems [10], it acts as a pre-biotic ferment in the colon by the probiotic microflora and produces short-chain fatty acids in maintaining gut health [11].

Roasting and coating are some of the best techniques for enhancing shelf life and sensory quality of flaked rice. Edible chocolate coating of roasted flaked rice (RFR) may find wider acceptability as breakfast snacks in comparison to the corn flakes, which contributes zein as an incomplete protein. Thus, in order to enhance the nutritional value of flaked rice, it can be coated with a mixture of dark chocolate, jaggery, guar gum, and milk powder. Jaggery is an important nutritive sweetener containing 65–85% sucrose as well as being a good source for minerals such as iron (11–12 mg/100 g), phosphorus (4 mg/100 g), and calcium (8 mg/100 g). The inclusion of jaggery in the diet is suggested to eradicate many diseases and promotes longevity [12]. Chocolate has a wide range of beneficial health effects as it is rich in antioxidants, flavonoids, and other important secondary metabolites [13]. Furthermore, chocolate is preferred by all age groups, especially children due to its distinctive flavor.

Coating materials are the semisolid materials that can be applied on the surface of RFR by the use of a rotating pan, a rotating drum, and a fluidized bed dryer [14]. The consistency of the coating materials may be controlled using the addition of hydrocolloids. Guar gum (*Cyamopsistetra gonoloba*) is a polysaccharide and is a water-soluble hydrocolloid used as an additive, emulsifier, stabilizer, and binding agent in processed food products to an extent of 0.5–1.0% [15]. The addition of guar gum adds dietary fiber, reduces the laxative requirement, and helps the person suffering from petulant bowel disorder [16]. Iron deficiency is the most common nutritional inadequacy resulting in the cause of anemia or impaired mental development in nearly one-third of the world’s population. Supplementation with ferrous sulfate (FeSO_4_) and folic acid as coating material improves the iron content in the coated product [17,18]. The physical parameters play a key role in forming the marketing significance of rice products [19].

Response surface methodology (RSM) is a statistical tool for composite variables. The RSM has a variety of advantages, one of which is the decrease in the number of experiments needed for assessment and analysis. RSM is a more efficient and speedier method of putting together research data than the traditional one-variable at a time or full-factor testing. The coefficient of determination for a well-fitted model should be always more than 80%. A higher R^2^ value, i.e., near unity, indicates that the empirical model is adequate for fitting the actual data, whereas a lower R^2^ value indicates that the model is ineffective for describing the relationship between variables. The dominant population uses rice as a staple food and roasting and coating with such a combination may act as an important vehicle to solve the associated nutritional problem through the development of widely acceptable RTE breakfast food items.

Our study is the first to utilize jaggery and dark chocolate in optimization and development of RTE rice-based functional breakfast food in conjunction with response surface methodology. It could explore the feasibility of iron fortification in rice-based functional breakfast as a rapid and cost-effective solution to iron deficiency anemia in economically disadvantaged populations where rice acts as major staple food. Iron fortification is considered to be the most cost-effective long term and convenient approach to provide additional absorbable iron. An attempt was also performed to add dark chocolate to RTE rice-based functional breakfast food as result of coating.

## 2. Materials and Methods

A variety of paddy (*Gurjari*) was obtained from Anand Agriculture University, India, for making flaked rice. Before the coating of flaked rice, roasting had been performed by adopting the developed optimized processing parameters [8]. Jaggery, dark chocolate, guar gum, ferrous sulfate (FeSO_4_), and skim milk powder for the coating ingredients were procured from the local market. The chemicals, reagents, and solvents used were analytical reagent grade. (Sigma-Aldrich Co. St. Louis, MO, USA).

### 2.1. Preparation of Chocolate—Coated Roasted Flaked Rice (CCRFR)

The RFR was coated with jaggery and dark chocolate at different level of combinations (Table 1) of the Central Composite Rotatable Design (CCRD) of RSM. Jaggery was used in the range of 6.38 to 10.62 g, whereas dark chocolate was used at a range of 17.93 to 32.07 g. The skim milk powder, guar gum, and ferrous sulfate were taken as fixed ranges, i.e., 1.0%, 0.5%, and 43.33 mg/100 g, respectively [17].

All the fixed ingredients were dissolved in 20 mL of distilled water at room temperature before mixing in the coating material. The RFR was coated with the help of a laboratory-scale continuous tablet coating machine (Macro Scientific Works Pvt. Ltd., Delhi, India). The temperature of the coating machine hot air blower was fixed at 70 ± 2 °C and the speed of the coating pan was set 60 rpm/min for 10 min. The calculated amount of coating material was sprinkled on the RFR for adequate coating in a rotating pan. The sample was taken out from a coating pan and placed in an oven (hot air) at 50 ± 2 °C for 2 h after the completion of the coating process.

### 2.2. Physicochemical Characteristics

Dimensional parameters such as breadth (B), length (L), and thickness (T) in millimeter (mm) were determined manually with a vernier caliper (Mitutoyo Corporation, Kanagawa, Japan). The geometric mean dimension (GMD) and the surface area (SA) were calculated [20].

The thousand kernel weight (TKW) in grams (g) was determined for the counted kernels using the electronic weighing balance (accuracy 0.001 g) (Ishida Co. Ltd., Kyoto, Japan). Bulk density (BD) was determined as the ratio of the mass of a sample to its occupied volume (kg/m^3^) [21].

The proximate composition of samples for crude protein (%), fat (%), ash (%), fiber (%), and moisture content (%) was determined by using the standard methods [22]. Carbohydrate percentage was estimated by the difference [23]. Minerals of samples were determined from the wet digested method [22]. The free fatty acids of RFR and CCRFR were determined according to the study of Ranganna [23].

### 2.3. Textural Characteristics

The hardness was evaluated by utilizing a Texture Analyzer (Stable Microsystems, Surrey, United Kingdom) positioned by employing the load cell of 50 kg using probe P5 (5 mm diameter stainless steel). Time versus compression force program was employed to compress single coated or uncoated RFR along with thickness. Conditions set for the test were pre-test speed of 2 mm/s, 80% strain, 5 g of trigger force, and post speed 10 mm/s. The value of maximum peak force in Newton (N) was considered as the hardness of the sample [24].

### 2.4. Functional Characteristics

Milk absorption capacity (MAC) was determined by taking a 1 g sample in a centrifuge tube with 10 mL milk at room temperature and kept for agitation in a magnetic stirrer for 1 h. The sample was centrifuged for 25 min at 3000 rpm [24]. The supernatant was decanted and evaporated on a pre-weighed dish. The left out filtrate was weighed for MAC calculation.

(1) $$MAC\;\left(\frac{g}{g}\right)=\frac{Total\;weight\;of\;gel}{Final\;dryweight\;of\;sample}$$

The total phenol content of samples was examined by adopting the modified method [25].

### 2.5. Optical Characteristics

The color difference (ΔE) was calculated (Equation (2)) from the measured optical parameters with Hunter Lab Chromameter (Konica Minolta Sensing, Osaka, Japan) considering optical parameters of flaked rice as a reference. The values of *L*, *a*, and *b* were obtained straightforwardly from the colorimeter and L signifies the lightness (black towards white), a signifies redness (green towards red), and b signifies yellowness (blue towards yellow).

(2) $$\Delta E=\sqrt{{\left(L-L_{0}\right)}^{2\;}+{\left(a-a_{0}\right)}^{2}+{\left(b-b_{0}\right)}^{2}\;}$$

### 2.6. Fourier Transform Infra-Red Spectroscopy (FTIR)

Two mg of ground sample was mixed with 50 mg of potassium bromide (KBr) for making thin pallets for analysis by using a FTIR Spectrometer (Shimadzu Corporation, Tokyo, Japan). The instrument was calibrated with a KBr pellet. The spectra were noted at a resolution of 2 cm^−1^ and intensity between the range of 4000–400 cm^−1^.

### 2.7. Pasting Properties

Viscosity properties of the RFR and CCRFR were studied by utilizing the instrument Rapid Visco Analyzer (RVA, Newport Scientific, Melbourne, Australia). The pasting properties were determined with the method followed by Kumar and Prasad [26].

### 2.8. Morphological Characteristics

Morphology of RFR and CCRFR was examined by Scanning Electron Microscope (SEM, Jeol, Tokyo, Japan). Double-sided tape was utilized and the samples were placed on different aluminum stubs, coated with gold, and then transferred to the instrument to obtain the different micrographs at variously specified magnifications.

### 2.9. Sensory Characteristics 

Sensory evaluation was carried out to determine the quality of the samples on color, texture, taste, and overall acceptability via a 9 points hedonic scale with a trained panel comprised of 25 members [23]. All panelists were given a glass of water to drink throughout the sensory analysis and were instructed to rinse their mouths and discard the water between each sample’s examination. The panelists expressed their opinions on samples by assigning a score to each of the attributes on a 9 point hedonic scale where 9 was ‘like extremely’ and 1 was ‘dislike extremely’ [27].

Panelists represent both male and females whose age varies from 25 to 48. All of the panelists were trained and chosen based on their enthusiasm and motivation, food attitudes, health, and understanding of ready to eat snack foods.

### 2.10. Experimental Design and Optimization

Statistical optimization was carried out by using RSM as a tool. Two independent variables (k = 2) at 5 levels of CCRD were adopted. Jaggery and dark chocolate were fixed to different combination levels in coded as well as un-coded form (Table 1). Five (5) different levels in coded form for each experiment were −α, −1, 0, +1, and +α, where α = (2) k/4 = (2) 1/2 = 1. 414. Thirteen numbers of experimental runs were explored for four (4) axial points, four (4) factorial experimental points, and five (5) experimental central points (Table 2) by using the following equation.

(3) $$Number\;\left(total\right)of\;experimental\;combinations=2^{k}+2^{*}k+experimental\;central\;points$$

The jaggery and dark chocolate ranges were chosen as per the preliminary experimental trials conducted. The lowest and highest level fixed for jaggery was 6.38 g and 10.62 g, respectively. Similarly, the lowest level for dark chocolate was 17.93 and the highest level was 32.07 g. The effect of varying the jaggery and dark chocolate on the dependent variables was observed and analyzed statistically by using Stat-Ease software (Stat-Ease 6.01, Stat-Ease Inc., Minneapolis, MN, USA).

A II order polynomial equation (Equation (4)) was fixed to the experimental values of dependent variables to observe the effects of selected independent variables in the following:

(4) $$Y_{a}=\beta a0+\sum_{i=1}^{n}\beta_{ai}x_{i\;}+\sum_{i=1}^{n-1}\sum_{j=i+1}^{n}\beta_{aij}x_{i\;}x_{j\;}+\sum_{i=1}^{n}\beta_{aii}x_{i}^{2}$$

where, *β_a0_* represents fitted response value at the design’s center point; *β_i_, β_ij_*, and *β_ii_* are linear, interface, and quadratic regression coefficients; *Y_a_,* is dependent variable; *x* is an independent variable; and *x_i_* represents the coded form of independent variables (*x*_1_ as jaggery and *x*_2_ as dark chocolate).

The independent variables of coating materials jaggery and dark chocolate were optimized numerically. All the values of independent variables were reserved in range whereas the dependent variables, i.e., responses be fixed minimized, maximized, and in range, depended on the desired attributes for developing highly acceptable CCRFR. Multiple response optimizations as a tool were applied for obtaining the best combination of independent variables based on the selected response of variables conditions; sensory scores for all the attributes were the maximum and the hardness in the final product was used as the minimum [28].

### 2.11. Statistical Evaluation

ANOVA (One-way analysis of variance) was utilized for the evaluation of experimental records with Statistica.v.12. (StatSoft India Pvt. Ltd., New Delhi, India) and XLSTAT (Addinsoft, New York, NY, USA). The levels of independent variables, jaggery, and dark chocolate in coating material composition were evaluated by Statistica.v.12. Values expressed as a mean ± SD were used to show the significant differences among the samples at the significance level of *p* ≤ 0.05 [29].

## 3. Results and Discussion

### 3.1. Preparation of CCRFR

Flaked rice is pre-cooked, possesses low moisture content, is shelf-stable, is an intermediate food product, and is commonly used for the preparation of various snack food items. RFR is one of the RTE food products consumed after seasoning or directly with the addition of milk and curd. The milk absorption characteristics of RFR play an essential role in the eating quality and acceptability as a commercial breakfast food item [8]. In order to quash the linked limitation of the one-variable-at-a-time (OVAT) method, the statistical optimization approach of CCRD of RSM was applied. The levels of skim milk powder (1.0%), guar gum (0.5%), and ferrous sulfate (43.33 mg/100 g) were taken as constant. The effect of jaggery (6.38 to 10.62 g) and dark chocolate (17.93 to 32.07 g) was observed on physical, textural, functional, optical, and sensory characteristics to optimize the CCRFR.

### 3.2. Physicochemical Characteristics

**Figure 1:**
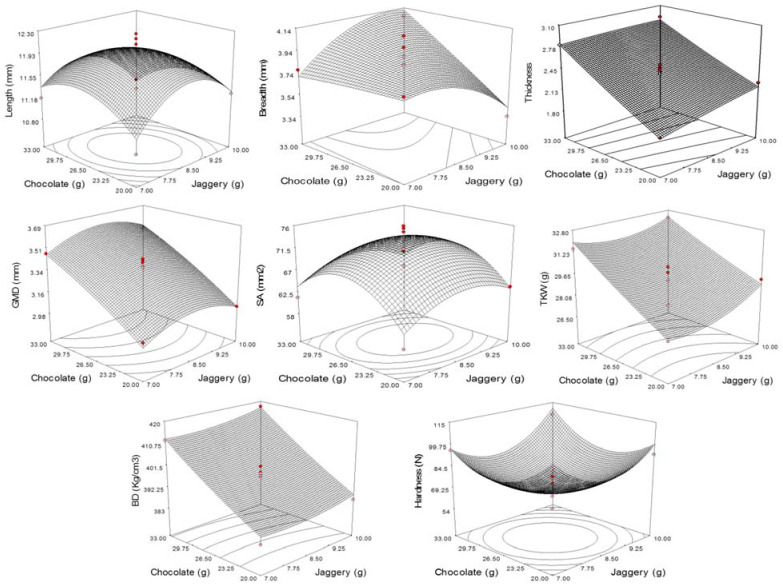
Physical and textural responses as affected by the levels of jaggery and chocolate.

Dimensional and gravimetric properties are important components of physical properties. The significance of physical properties in various applications is well documented. These parameters are needed for the design of types of equipment sorting, handling, processing, transportation, and storage of food materials. The dimensional parameters such as length, breadth, and thickness of CCRFR were observed to vary from 10.83 mm to 12.17 mm, 3.34 mm to 4.11 mm, and 1.89 mm to 3.02 mm, respectively, upon varying the levels of jaggery and dark chocolate (Table 2). The highest values of breadth (4.11 mm) and thickness (3.01 mm) for the CCRFR were observed at experiment no. 8, where the maximum amount of dark chocolate (32.07 g) was used with the jaggery (8.50 g) at the assumed optimal level (Table 2). The effects of jaggery and dark chocolate on dimensional parameters are shown (Figure 1).

Regression data for dimensional parameters against jaggery and dark chocolate in CCRFR produced the following II-order polynomial model:

(5) $$Length=12.00-0.042A-4.31B-0.15AB-0.49A^{2}-0.33B^{2}$$

(6) $$Breadth=3.91+0.013A+0.16B+0.19AB-0.14A^{2}-0.11B^{2}$$

(7) $$Thickness=2.44+0.079A+0.39B-0.052AB-0.013A^{2}-0.021B^{2}$$

where *A* is the amount of jaggery and *B* is the amount of dark chocolate.

The significance of constant and coefficient of the developed model based on ANOVA are presented in Table 3, which shows that the coefficients of multiple regression values were less than 80.0% for length but more for breadth and thickness. The low values of chi-square, percent error, and root mean square error (RMSE) represents the adequacy of a model for their use in the prediction of values. Analysis of the coefficients of the second-order model further showed that amount of dark chocolate had a significant linear positive effect at *p* ≤ 0.1 on breadth and thickness of CCRFR.

#### 3.2.1. Geometric Mean Diameter (GMD) and Surface Area (SA)

The GMD and SA of CCRFR varied from 2.98 to 3.69 mm and 58.30 to 75.91 mm^2^, respectively, for different concentrations of coating materials (Table 2). The highest and lowest value of GMD were found when the respective highest (35.69 g) and lowest (17.93 g) amounts of dark chocolate with jaggery at the level of 8.50 g were utilized. The variations in GMD may be due to the extent of material deposition during coating depending on the amount of dark chocolate present in the coating material. The SA was found to be governed by the length of the CCRFR and respectively found highest (75.91 mm^2^) when length (12.25 mm) was highest at experiment no. 12 and lowest at experiment no. 1 (Table 2).

The regression of data for GMD and SA of CCRFR against jaggery and dark chocolate levels produced the following II-order polynomial equations.

(8) $$GMD=3.39+0.02A+0.24B+2.24AB-0.11A^{2}-0.03B^{2}$$

(9) $$SA=73.07+0.82A+0.98B-0.26AB-3.65A^{2}-6.14B^{2}$$

The significances of the model constant and coefficient based on ANOVA of II order polynomial model for GMD and SA are provided in Table 3. The response surface plot of GMD and SA (Figure 1) also shows that dark chocolate is positively affecting the GMD, whereas SA is affected by the amount of jaggery and dark chocolate at the quadratic level (Table 3). The coefficient of multiple determinations (R^2^) shows that the model explained 98.4 and 81.7% variability for GMD and SA, respectively. The effect of SA on coated popcorn was reported by Sumonsiri and Barringer [30].

#### 3.2.2. Thousand Kernel Weight (TKW)

TKW, which is a significant gravimetric parameter, was observed to vary from 26.58 to 32.72 g when the amount of jaggery and dark chocolate varied in the specific combinations as per CCRD of RSM (Table 2). The utmost and least values of TKW were found to be associated with the extent of dark chocolate quantity used in the coating material as reflected in governing the GMD. The response surface plot for TKW as a function of coating materials has been shown (Figure 1). The effect of independent variables on TKW could also be explained by the quadratic model (Equation (10)).

(10) $$TKW=29.35+0.48A+1.97B-0.31AB+0.67A^{2}+0.12B^{2}$$

The coefficient of the above model shows that dark chocolate (Table 3) is significantly (*p* ≤ 0.01) and positively affecting the TKW. The low values of chi-square (0.210), % Error (0.058), and RMSE (0.690) with a higher value of R^2^ (85.4%) shows that variation in the TKW ratio is predominantly due to changes in dark chocolate level. The density of used ingredients of coating materials has enhanced the TKW of CCRFR [31].

#### 3.2.3. Bulk Density (BD)

The quality of food materials can be evaluated based on gravimetric characteristics. BD can be calculated by measuring the mass and bulk volume occupied by the material. BD of CCRFR ranged from 383.00 to 419.72 kg/cm^3^ for different combinations of jaggery and dark chocolate (Table 2). The utmost value of BD was observed at experiment no. 8 where the highest quantity of dark chocolate was used in the coating material. The applied dry heat during roasting and subsequent compression on flaking has resulted in decreased BD [8]. Furthermore, the coating has resulted in enhanced TKW and thus increases the BD of CCRFR [32]. The sign and magnitude of the regression coefficient of jaggery and dark chocolate had a significant and positive effect at the linear level of jaggery and dark chocolate on the BD of CCRFR (Table 3). The value of BD as dependent on the amount of jaggery and dark chocolate is presented (Equation (11)).

(11) $$BD=398.53+1.88A+13.66B+0.36AB+1.70A^{2}+1.15B^{2}$$

The coefficient of multiple determinations (R^2^) as 97.2% with low values of chi-square (0.114), % Error (0.002), and RMSE (1.852) showed the applicability of the developed model in predicting the result.

### 3.3. Textural Characteristics

Hardness is an important textural parameter defined as the maximum force required in the deformation of compressing food products. The hardness of the RFR varied in the range from 54.07 N to 114.35 N (Table 2). The sign of regression coefficient and magnitude of coating materials jaggery and dark chocolate demonstrated the significant (*p* ≤ 0.01) positive effect at a quadratic level on the hardness of CCRFR (Table 3). The maximum hardness of CCRFR was found when the highest amount of jaggery (10.62 g) and dark chocolate (25.00 g) at an assumed optimum level was used (Figure 1). The surface cementing of void space or cracks of RFR on coating has resulted in the enhanced level of hardness in CCRFR [33]. Coefficients, constant, and model parameters with statistical findings are presented in Table 3. Hardness values dependent on the amount of jaggery and dark chocolate are presented through the full quadratic model (Equation (12)).

(12) $$Hardness=68.94+2.21A+0.47B+4.84AB+17.66A^{2}+16.75B^{2}$$

The lower values of chi-square (7.65), RMSE (6.616), and percent error (0.87) for the developed model of hardness were observed. Furthermore, R^2^ has reflected the variability of 86.6%.

### 3.4. Functional Characteristics

**Figure 2:**
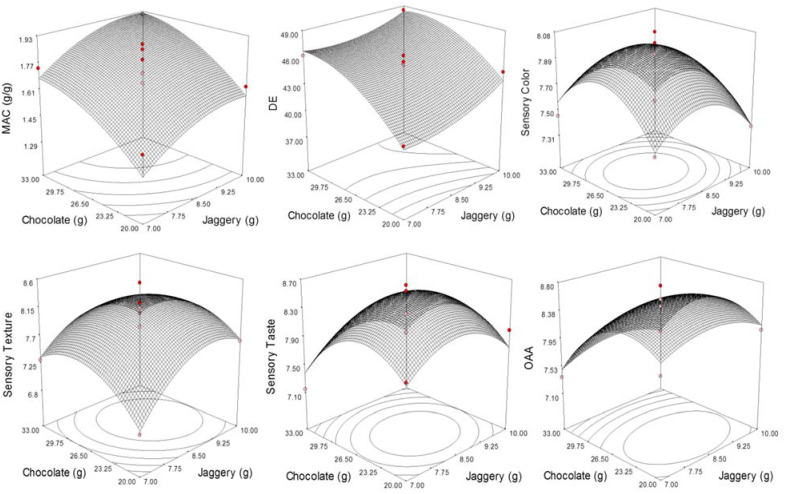
Functional and sensory responses as affected by the levels of jaggery and chocolate.

Loss of crispness of roasted food on coming in contact with water is the major hindrance in making the commercially acceptable food product. The process of coating has resulted in a significant decrease in the rate of milk absorption and thus loss of crispness was delayed considerably. The MAC of CCRFR varied from 1.29 to 1.91 g/g for different concentrations of coating materials (Table 2). However, the absorption of milk was found to be significantly affected and linearly dependent on the quantity of jaggery and dark chocolate used (Table 3). The highest value milk absorption was observed at experiment no. 4 with jaggery (10.00 g) as well as dark chocolate (30.00 g). MAC was found lowest when the lowest quantity of dark chocolate (17.93 g) was used (Figure 2). Coating materials used formed a smooth layer on the surface and filled the gap and void spaces and, thus, reduced the MAC and enhanced the milk absorption time [34]. The data of MAC as dependent on the amount of jaggery and dark chocolate is provided in Equation (13).

(13) $$MAC=1.78+0.12A+0.17B+1.25AB-0.073A^{2}-0.072B^{2}$$

The value of R^2^ reflects realistic concord by showing 83.2% variability. The low values of percent error (0.052) and chi-square (0.258) further confirm the applicability of the model for MAC.

### 3.5. Optical Characteristics

Food products are initially accepted based on their optical characteristics. Optical properties reflect the effect of raw ingredients on the prepared food products and various changes occurred during processing and storage. Thus, keeping the color parameters of RFR as a reference, the ΔE was determined by following Equation (2) and observed to range from 37.40 to 48.48. An increase in the ΔE may be attributed to the addition of a dark compound of coating material on the surface of CCRFR [35]. The least value of ΔE was observed when the smallest quantity of coating materials such as jaggery (17.31) and dark chocolate (8.50) was applied and vice versa.

The value of ΔE dependent on the amount of jaggery and dark chocolate is presented through Equation (14).

(14) $$\Delta E=45.57+1.16A+2.80B-0.37AB+0.89A^{2}-1.69B^{2}$$

The magnitude and sign of the regression coefficient of coating materials demonstrated the level of significance (Table 3). As expected, these have reflected the positive effect on ΔE of CCRFR (Figure 2). A higher value of the coefficient of multiple determinations reflects 96.8% variability with lower values of chi-square, RMSE, and percent error which further supports the developed model adequacy for predicting the ΔE data of CCRFR (Table 2).

### 3.6. Sensory Characteristics

Color, texture, taste, and overall acceptability (OAA) scores of CCRFR varied from 7.31 to 8.08, 6.85 to 8.54, 7.15 to 8.62, and 7.18 to 8.75, respectively (Table 2). The minimum color score was found at experiment no. 4, where a higher amount of jaggery (10.00 g) and dark chocolate (30.00 g) in the coating materials were used. At this level, it was found to have the highest MAC (1.91 g/g) and color difference (48.48). Experiment no. 13 produced the highest color score of 8.08. This combination of jaggery and dark chocolate has also resulted in the highest sensory scores for texture, taste, and OAA. The lower amount of jaggery with a lower and higher amount of dark chocolate resulted in the lower sensory texture and taste score, respectively (Figure 2). On the other side, the use of the highest level of dark chocolate had adversely affected the OAA and resulted in the lower score. A minimum score of seven for color, texture, taste, and hence of OAA score was a well-thought-out essential in evaluating the assortment of a grade of ingredients for CCRFR.

The sensory figures of CCRFR were fixed with II-order polynomial model and the equations obtained were as follows.

(15) $$Color=7.98-0.070A+0.018B-0.019AB+0.23A^{2}-0.29B^{2}$$

(16) $$Texture=8.20+0.23A+0.089B-0.13AB-0.43A^{2}-0.41B^{2}$$

(17) $$Taste=8.52-0.017A-0.17B+0.038AB-0.40A^{2}-0.51B^{2}$$

(18) $$OAA=8.57+0.018A-0.32B+0.023AB-0.22A^{2}-0.49B^{2}$$

The coefficient of multiple determinations (R^2^) for color, texture, taste, and overall acceptability scores were 94.2%, 91.3%, 89.1%, and 94.5%, respectively (Table 3). These values showed that the developed models elucidated more than 90% variability. Values of R^2^ greater than 80% are better for elucidating the variability in a model [21], whereas the value of 85% for R^2^ is better for the elucidation of difference in the case of sensory figures [36]. Percent error, lower values of RMSE, and chi-square further support the adequacy of the developed model. Therefore, the model developed to envisage sensory scores for CCRFR with various ingredient levels was found as sufficient.

Coefficients of a full II-order polynomial model for sensory scores and the significance of each term in the model are presented in Table 3. The table shows that jaggery has a significant role in controlling the color and texture component of the developed product, while dark chocolate controls the sensory taste and OAA part at a linear level. Jaggery and dark chocolate are found to have a significant role at the quadratic level but significant interaction effects on sensory scores were not observed.

### 3.7. Optimization of CCRFR

**Figure 3:**
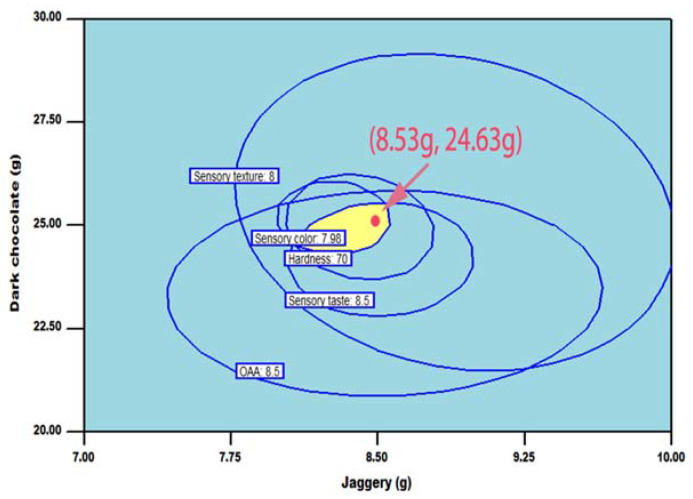
Superimposed counter plots for sensory and textural responses as affected by the levels of jaggery and chocolate.

In the experimental series, all independent variables reserved were in range; although based on CCRFR desirable characteristics, responses were fixed in the combination of range, maximum, and minimum for multiple response optimizations. The following limits were planned: All the sensory attributes were maximized while hardness for the finalization of the optimized product was minimized by keeping all other variables under range. The generated optimum levels of independent variables, i.e., jaggery 8.53 g having the corresponding coded value of 0.02 and 24.63 g dark chocolate having the corresponding coded value of −0.07, are the most desirable solution in the numerical optimization approach to obtain the optimum CCRFR. The obtained optimized combination of ingredient levels was verified by using the graphical optimization technique (Figure 3). The high quality of CCRFR could be prepared with the various combinations in graphical optimization technique depending on the choice of different parameters other than selected parameters such as cost, availability, and ease in handling [37]. The obtained theoretical results for the physical, textural, functional, optical, and sensory characteristics were confirmed by preparing the CCRFR of numerically optimized coating materials while characterizing the developed highly acceptable RTE functional food product. 

### 3.8. Comparison of Optimized CCRFR to RFR

#### 3.8.1. Physical Characteristics 

Changes in the physical properties of CCRFR from RFR and estimated theoretical values as determined through numerical optimization are presented in Table 4. The length, breadth, and thickness of RFR increased upon coating, i.e., 12.43 ± 0.78 to 12.52 ± 0.29 mm, 4.09 ± 0.08 to 4.24 ± 0.33 mm, and 2.30 ± 0.09 mm to 2.39 ± 0.01 mm, respectively. 

The GMD and SA of the coating of RFR were found to be slightly affected due to the combined effect of dimensional changes. An increased TKW of RFR was observed after coating mainly due to material deposition and varied from 23.13 ± 0.16 to 29.44 ± 0.41 g, whereas the BD increased from 364.15 ± 6.24 to 392.72 ± 4.02 kg/m^3^. The combined effect of changes in the dimensional characteristic and gravimetric characteristics coating with jaggery and dark chocolate had entirely changed the material characteristics.

#### 3.8.2. Chemical Characteristics

The proximate and mineral compositions of RFR along with CCRFR are compared (Table 5). The total moisture content of RFR and CCRFR was observed to be increased from 4.61 ± 0.09% to 6.72 ± 0.64% due to the associated characteristics of applied coating ingredients. The total percentage of the fat content of CCRFR was found greater than RFR and varied from 2.81 ± 0.15 to 10.16 ± 0.03%. The increase in the fat content might be attributed to the addition of dark chocolate, which had a high amount of fat content (42.63%). The crude protein, fiber, and ash contents were found to change from 6.10 ± 0.05 to 8.12 ± 0.08%, 1.84 ± 0.13 to 2.61 ± 0.12%, and 1.58 ± 0.05 to 2.05 ± 0.13%, respectively. Minerals contents were found to be significantly increased in CCRFR and they consist of iron (1.64 ± 0.19 to 26.73 ± 0.12 mg/100 g), calcium (19.29 ± 0.62 to 51.59 ± 0.09 mg/100 g), sodium (5.40 ± 0.36 to 17.81 ± 0.51 mg/100 g), and potassium (119.00 ± 1.73 to 349.35 ± 2.25 mg/100 g). The minerals contents were found higher in CCRFR because of the addition of jaggery and ferrous sulfate at a constant level with the coating material. The increase in other nutrients in CCRFR has resulted in the lowering of carbohydrate content. The free fatty acid content was found to be 0.17 ± 0.06% in CCRFR (Table 5). 

#### 3.8.3. Fourier Transform Infrared Spectroscopy (FTIR)

**Figure 4:**
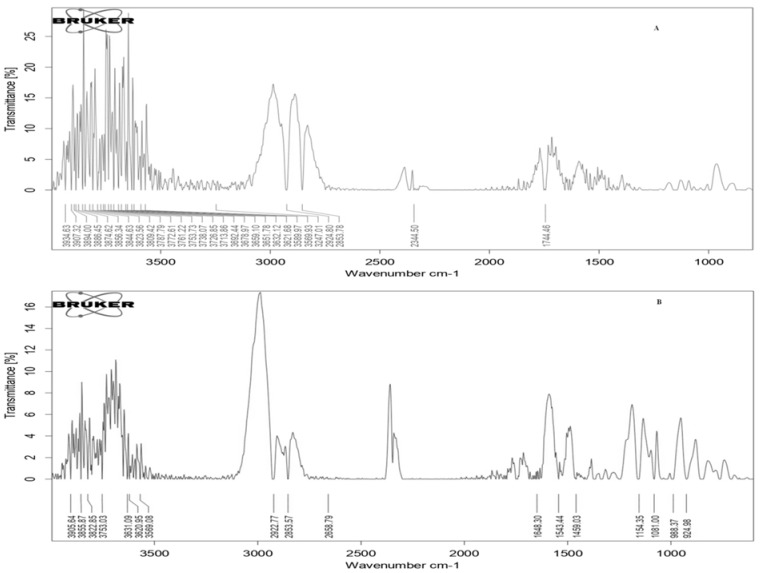
Fourier Transform Infrared Spectroscopy spectra of (**A**) RFR and (**B**) CCRFR.

FTIR is a spectroscopic technique used to investigate the infrared (IR) spectrum of absorption or emission of the subjected sample. IR absorption spectra imitate absorption bands after various available functional groups. The FTIR spectra of RFR and CCRFR are depicted in Figure 4. Bands obtained at 924.98 cm^−1^ wavelength and 980.41 cm^−1^ wavelength reveal the existence of functional groups such as –C–O and –C=O. The obtained visible spectrum scale of RFR and CCRFR had no major changes in between bands 924.98 cm^−1^ and 1543.44 cm^−1^ bands. The group array 1648.30–1744.50 cm^−1^ showed the existence of fatty acids in RFR and CCRFR. Similar results were also observed by Vemireddy et al. [38]. The absorption band at a 2853.57 cm^−1^ wavelength to 2924.50 cm^−1^ wavelength indicated the regular and irregular –CH_2_– stretch band, while the –O–H– bond stretching was linked at 3620.95 cm^−1^ wave number to 3822.55 cm^−1^ wave number. The shift of band from RFR to CCRFR wave number, i.e., 1648.30 cm^−1^ to 1744 cm^−1^ and 2410.44 cm^−1^ to 2344.50 cm^−1^ after the coating, showed 95.7 cm^−1^ shift towards the higher side and 65.94 cm^−1^ wave number towards the lower side in CCRFR. The O-H stretch band was observed at 3823.55 cm^−1^ wavelength and 3664.13 cm^−1^ wavelength in CCRFR. No major difference was found between the bands compared to RFR except for some stretching. After applying the coating on RFR, no key variation was found in bands as revealed in the IR spectrum [39].

#### 3.8.4. Pasting Properties

**Figure 5:**
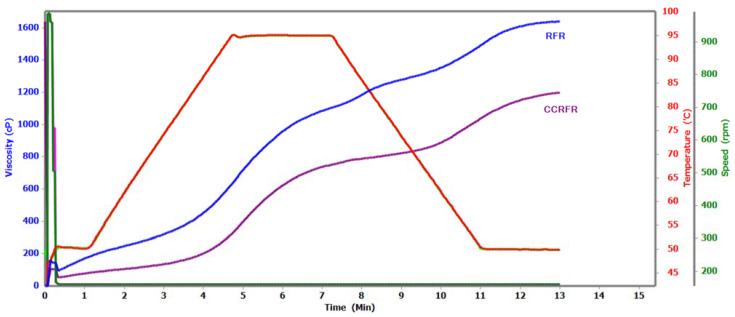
Cross sectional (CS) view and micrograph of uncoated (RFR) and coated roasted flaked rice (CCRFR).

Pasting properties exhibited a potential method to represent the attributes of starchy food products. The pasting characteristics of RFR on coating to CCRFR were estimated using RVA. The effects of coating ingredients on RFR the pasting activities were evaluated (Figure 5). The peak viscosity was noted to be higher (1086.00 cP) than CCRFR (737.00 cP). The related trends have been noted for the trough, break down, and final viscosities. The setback viscosity was found highest in CCRFR (565.00 cP) (Table 5). Final viscosity rise of RFR during chilling may be due to raise the water griping capacity on composite of starch particles. The addition jaggery contains sucrose and the sucrose acts as an anti-plasticizer effect on CCRFR starch granule. The similar results were noted by Kose [40] and Sharma et al. [41]. They described the starch and sucrose matrix which inhibit the water amalgamation ability and cannot enlarge up to the requisite suspension. The addition of dark chocolate results in the increase in the fat composition in CCRFR, which decreases the peak and final viscosity of CCRFR. Moreover, it may be due to fatty acids restricting the starch granule from hydration and swelling, which was determined by Zhou et al. [42]. Therefore, CCRFR has less water holding capacity compared to RFR. This may be due to the application of coating ingredients on RFR and the decrease in the amount of starch granules. 

#### 3.8.5. Functional and Textural Characteristics

The MAC of the RFR and optimized CCRFR was found to decrease from 3.63 ± 0.35 to 1.59 ± 0.43 g/g (Table 5). The decrease in MAC may be attributed to the packing of cracks and pores of RFR with the coating materials. It was also observed that due to coating, the milk absorption time for the loss of crispness was extended. On the other hand, the coating has not much affected the hardness values of optimized products (Table 4) and this may be attributed to the increased moisture content of coated product from 4.61 to 6.72% (Table 5).

Phenolic compounds behave as an antioxidant as these possess redox properties. The phenolic content of the RFR and CCRFR was 1.310 ± 0.044 and 91.203 ± 1.290 mg gallic acid equivalent/100 g, respectively (Table 5). The reason might be because the coating ingredients, i.e., dark chocolate and jaggery, are a good source of polyphenols.

#### 3.8.6. Morphological Characteristics (SEM)

**Figure 6:**
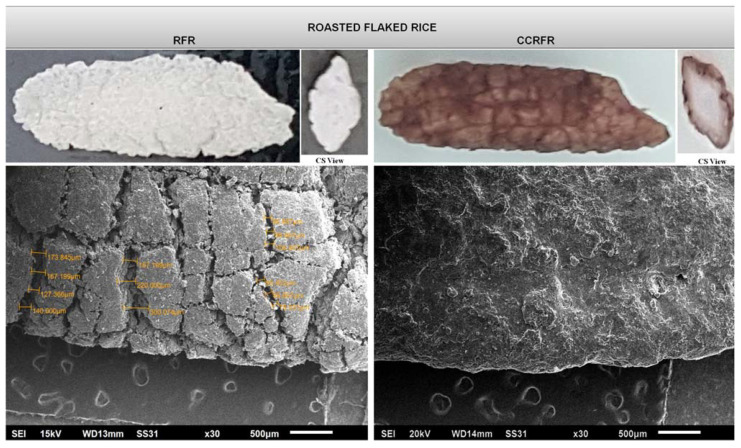
Micrographs of RFR and CCRFR.

A SEM is an instrument used to study the outer morphological characteristics of the product and its internal microstructure [26]. The structures of RFR and CCRFR were shown to be widely varied at the micron level (Figure 6). The surface of RFR was rough with several cracks and about average size of 185.086 µm, whereas the surface of CCRFR becomes almost smooth and cracks present on the outer surface are filled because of the coating. Coating materials act as a filler to fill the gaps present on the surface of the CCRFR and thus improved the characteristics of the developed product and can be used as a suitable alternative to the existing corn-based breakfast food items.

#### 3.8.7. Sensory Characteristics

The sensory properties of the optimized CCRFR resembled the estimated values of sensory scores obtained by numerical optimization (Table 4). Sensory scores for the color, texture, taste, and OAA of optimized CCRFR were found to be 8.08 ± 0.09, 8.12 ± 0.12, 8.55 ± 0.13, and 8.62 ± 0.10, respectively, while the estimated respective sensory scores were 7.98, 8.20, 8.54, and 8.59, which were found well within the respective sensory ranges.

## 4. Conclusions

The coating of RFR with coating materials has significantly affected the studied physical, pasting textural, functional, optical, and sensory characteristics designed based on the experimentation of RSM. A combination of 8.53 g jaggery and 24.63 g dark chocolate was found to be the most desirable for the development of CCRFR. MAC decreased sustained crispiness. The best sensory attributes for the color, texture, taste, and overall acceptability of optimized CCRFR were also improved. Higher values of all the sensory attributes for the developed CCRFR with improved morphological properties may find its place among the highly acceptable RTE food product and can be used as breakfast food items and as a suitable dietary source for celiac subjects in line with gluten-free food.

## Figures and Tables

**Table 1:** Central composite rotatable design in coded and uncoded forms of independent variables.

Independent Variable	Code	Levels in Coded Form				
		−1.414	−1.000	0.000	+1.000	+1.414
Jaggery, g	X_1_	6.38	7.00	8.50	10.00	10.62
Chocolate, g	X_2_	17.93	20.00	25.00	30.00	32.07

**Table 2:** Physical, textural, functional, and optical sensory characteristics of CCRFR as affected by the levels of jaggery and chocolate.

Exp. No.	Independent Variables		Dependent Variables *													
			Physical, Textural, Functional and Optical Characteristics										Sensory Characteristics			
	Jaggery	Dark Chocolate	L (mm)	B (mm)	T (mm)	GMD (mm)	SA (mm^2^)	TKW (g)	BD (kg/cm^3^)	HD (N)	MAC (g/g)	ΔE	Colour	Texture	Taste	OAA
	X_1_	X_2_	Y_1_	Y_2_	Y_3_	Y_4_	Y_5_	Y_6_	Y_7_	Y_8_	Y_9_	Y_10_	Y_11_	Y_12_	Y_13_	Y_14_
1	−1	−1	10.83	3.81	1.93	3.04	58.30	27.30	383.00	102.87	1.47	40.76	7.46	6.85	7.85	8.00
2	1	−1	11.25	3.34	2.24	3.04	63.71	29.28	386.88	93.13	1.63	44.46	7.38	7.62	8.00	8.09
3	−1	1	11.17	3.77	2.81	3.47	61.44	31.47	412.27	95.85	1.74	46.26	7.46	7.31	7.15	7.36
4	1	1	11.00	4.06	2.91	3.49	65.80	32.22	417.57	105.46	1.91	48.48	7.31	7.54	7.46	7.55
5	−1.414	0	11.33	3.54	2.31	3.11	67.86	30.37	401.31	102.25	1.36	45.94	7.69	7.08	7.85	8.27
6	1.414	0	10.92	3.74	2.47	3.23	65.58	31.13	405.47	114.35	1.79	48.31	7.46	7.69	7.62	8.18
7	0	−1.414	11.50	3.70	1.89	2.98	60.82	26.58	384.83	107.03	1.29	37.40	7.38	7.31	7.54	8.18
8	0	1.414	11.42	4.11	3.02	3.69	62.66	32.72	419.72	105.91	1.86	46.54	7.54	7.54	7.46	7.18
9	0	0	11.33	3.82	2.46	3.37	67.94	29.23	401.33	77.42	1.71	45.23	7.92	7.85	8.23	8.55
10	0	0	12.17	3.97	2.51	3.42	74.73	30.19	398.86	54.07	1.65	46.27	8.00	8.23	8.62	8.45
11	0	0	12.08	3.89	2.40	3.36	75.41	30.18	396.92	63.14	1.79	45.38	8.00	8.23	8.62	8.55
12	0	0	12.25	4.07	2.38	3.43	75.91	29.77	397.22	72.55	1.88	45.39	7.92	8.15	8.62	8.75
13	0	0	12.17	3.81	2.44	3.40	71.37	27.37	398.33	77.53	1.85	45.57	8.08	8.54	8.54	8.55

* L—Length; B—Breadth; T—Thickness; GMD—Geometric mean dimension; SA—Surface area; TKW—Thousand kernel weight; BD—Bulk density; HD—Hardness; MAC—Milk absorption capacity; ΔE—Color difference; OAA—Overall acceptability.

**Table 3:** Regression coefficients of fitted models with statistical parameters.

	Physical, Textural, Functional and Optical Characteristics										Sensory Characteristics			
Parameters	L (mm)	B (mm)	T (mm)	GMD (mm)	SA (mm^2^)	TKW (g)	BD (kg/cm^3^)	HD (N)	MAC (g/g)	ΔE	Colour	Texture	Taste	OAA
**Constant**	12.00	3.911	2.439	3.394	73.071	29.347	398.533	68.943	1.776	45.567	7.985	8.200	8.523	8.569
**X_1_**	−0.042 ^ns^	0.013 ^ns^	0.079 *	0.023 ^ns^	0.820 ^ns^	0.476 ^ns^	1.883 *	2.124 ^ns^	0.116 **	1.159 ***	−0.070 *	0.234 **	0.017 ^ns^	0.018 ^ns^
**X_2_**	0.045 ^ns^	0.156 *	0.393 *	0.235 ***	0.978 ^ns^	1.972 ***	13.662 ***	0.466 ^ns^	0.170 ***	2.804 ***	0.018 ^ns^	0.089 ^ns^	−0.167 *	−0.325 ***
**X_1._X_2_**	−0.15 ***	0.189 *	−0.052 *	0.002 ^ns^	−0.264 ^ns^	−0.309 ^ns^	0.355 ^ns^	4.837 ^ns^	0.001 ^ns^	−0.370 ^ns^	−0.019 ^ns^	−0.135 ^ns^	0.038 ^ns^	0.023 ^ns^
**X_1_^2^**	−0.49**	−0.143 *	−0.013 ^ns^	−0.109 ***	−3.654 **	0.667 ^ns^	1.704 ^ns^	17.664 ***	−0.073 ^ns^	0.889 **	−0.233 ***	−0.427 ***	−0.396 ***	−0.222 ***
**X_2_^2^**	−0.33 ^ns^	−0.011 ^ns^	0.021 ^ns^	−0.028 *	−6.145 ***	0.118 ^ns^	1.147 ^ns^	16.749 ***	−0.072 ^ns^	−1.688 ***	−0.290 ***	−0.408 ***	−0.512 ***	−0.495 ***
**R^2^**	0.749	0.861	0.986	0.984	0.817	0.854	0.972	0.866	0.832	0.968	0.942	0.913	0.891	0.945
**Chi square**	0.065	0.020	0.008	0.003	1.087	0.210	0.114	7.653	0.052	0.076	0.007	0.031	0.044	0.019
**% E**	0.044	0.044	0.026	0.006	0.124	0.058	0.002	0.870	0.258	0.017	0.007	0.030	0.043	0.018
**RMSE**	0.243	0.077	0.038	0.026	2.399	0.690	1.852	6.716	0.079	0.507	0.065	0.140	0.163	0.110

L—Length; B—Breadth; T—Thickness; GMD—Geometric mean dimension; SA—Surface area; TKW—Thousand kernel weight; BD—Bulk density; HD—Hardness; MAC—Milk absorption capacity; ΔE—Color difference; OAA—Overall acceptability. Level of significance *** *p* < 0.01%, ** *p* < 0.05%, * *p* < 0.1%, ^ns^ non-significant.

**Table 4:** Comparative physical, textural, functional, optical, and sensory properties of RFR and CCRFR.

Parameters	RFR	CCRFR	
		Estimated Value	Actual Value
L (mm)	12.43 ± 0.06 ^a^	12.00	12.52 ± 0.29 ^a^
B (mm)	4.09 ± 0.08 ^b^	3.90	4.24 ± 0.33 ^a^
T (mm)	2.30 ± 0.09 ^a^	2.41	2.39 ± 0.01 ^a^
GMD (mm)	4.95 ± 0.08 ^b^	3.38	4.97 ± 0.05 ^a^
SA (mm^2^)	77.02 ± 2.61 ^a^	72.98	77.52 ± 1.43 ^a^
TKW (g)	23.13 ± 0.16 ^b^	29.21	29.44 ± 0.41 ^a^
BD (kg/m^3^)	364.15 ± 6.24	397.56	392.72 ± 4.02
HD (N)	62.48 ± 5.01 ^b^	69.05	75.02 ± 3.66 ^a^
MAC (g/g)	3.63 ± 0.35 ^a^	1.77	1.59 ± 0.43 ^b^
ΔE	2.39 ± 0.03 ^b^	45.37	45.85 ± 0.41 ^a^
Color	7.33 ± 0.02 ^b^	7.98	8.08 ± 0.09 ^a^
Texture	7.67 ± 0.03 ^b^	8.20	8.12 ± 0.12 ^a^
Taste	8.33 ± 0.04 ^a^	8.54	8.55 ± 0.13 ^a^
OAA	8.61 ± 0.06 ^a^	8.59	8.62 ± 0.10 ^a^

Results are expressed as mean values ± standard deviations. Means in a row with same superscripts are not significantly different (*p* < 0.05). RFR—Roasted Flaked Rice; CCRFR—Chocolate Coated Roasted Flaked Rice; L—Length; B—breadth; T—thickness; GMD—geometric mean dimension; SA—surface area; TKW—thousand kernel weight; BD—bulk density; HD—hardness, MAC—milk absorption capacity; ΔE—color difference; OAA—Overall acceptability.

**Table 5:** Comparative chemical and pasting characteristics of RFR and CCRFR.

Parameters	RFR	CCRFR
Moisture (%)	4.61 ± 0.09	6.72 ± 0.64
Crude fat (%)	2.81 ± 0.15	10.16 ± 0.03
Crude fiber (%)	1.84 ± 0.13	2.61 ± 0.12
Protein (%)	6.10 ± 0.05	8.12 ± 0.08
Total ash (%)	1.58 ± 0.05	2.05 ± 0.13
Carbohydrate (%)	82.28 ± 0.21	71.11 ± 0.78
Energy (kcal/100 g)	387.09 ± 1.63	417.17 ± 1.43
Iron (mg/100 g)	1.64 ± 0.19	26.73 ± 0.12
Calcium (mg/100 g)	19.29 ± 0.62	51.59 ± 0.09
Sodium (mg/100 g)	5.40 ± 0.36	17.81 ± 0.51
Potassium (mg/100 g)	119.00 ± 1.73	349.35 ± 2.25
Total polyphenol (mg GAE/100 g)	1.31 ± 0.04	91.20 ± 1.29
Free fatty acid (%)	0.15 ± 0.03	0.17 ± 0.06%
**Pasting Properties**		
Peak viscosity (cP)	1086.00	737.00
Trough viscosity (cP)	1195.00	632.00
Break down viscosity (cP)	109.00	105.00
Final viscosity (cP)	1639.00	1197.00
Set back Viscosity (cP)	444.00	565.00
Peak time (min)	7.00	7.00
Pasting Temperature (°C)	88.80	95.20

Values are represented as mean ± standard deviation. RFR—Roasted Flaked Rice; CCRFR—Chocolate Coated Roasted Flaked Rice.
